# Altered Metabolites in the Occipital Lobe in Migraine Without Aura During the Attack and the Interictal Period

**DOI:** 10.3389/fneur.2021.656349

**Published:** 2021-05-20

**Authors:** Luping Zhang, Jinwen Huang, Zhengxiang Zhang, Zhijian Cao

**Affiliations:** ^1^Department of Radiology, Hangzhou TCM Hospital Affiliated to Zhejiang Chinese Medical University, Hangzhou, China; ^2^The First Clinical Medical College, Zhejiang Chinese Medical University, Hangzhou, China; ^3^Department of Neurology, The First Affiliated Hospital of Zhejiang Chinese Medical University, Hangzhou, China; ^4^Department of Radiology, The First Affiliated Hospital of Zhejiang Chinese Medical University, Hangzhou, China

**Keywords:** glutathione, choline, magnetic resonance spectroscopy, oxidative stress, inflammation, neuronal dysfunction

## Abstract

**Background:** Although there have been many magnetic resonance spectroscopy (MRS) studies of migraine, few have focused on migraines during an attack. Here, we aimed to assess metabolite changes in the brain of patients with migraine, both during an attack and in the interictal phase.

**Methods:** Six patients (one man and five women, mean age: 39 ± 10 years) with migraine without aura during the attack (MWoA-DA), 13 patients (three men and 10 women, mean age: 31 ± 9 years) with migraine without aura during the interictal period (MWoA-DI), and 13 healthy controls (HC) (four men and nine women, mean age: 31 ± 9 years) were studied. All subjects underwent an MRS examination focusing on the occipital lobe. Metabolite changes were investigated among three groups.

**Results:** The MWoA-DA patients had lower glutathione/total creatine ratio (GSH/tCr) than the MWoA-DI patients and HC. Furthermore, MWoA-DI patients showed lower total choline/total creatine ratio (tCho/tCr) than those in the other two groups. The GSH/tCr ratio was positively correlated with attack frequency in the MWoA-DI group. The tCho/tCr ratio was positively correlated with attack frequency and Migraine Disability Assessment Scale (MIDAS) scores in the MWoA-DA group.

**Conclusion:** The present study suggests the existence of distinct pathophysiological states between the MWoA-DA and MWoA-DI groups. Neuronal dysfunction is a possible predisposing factor for migraine attack onset, along with oxidative stress and inflammation.

## Introduction

Migraine is a common neurological disorder, characterized by recurrent headache and is typically accompanied by nausea, photophobia, and phonophobia (1). It affects a large proportion of the global population and causes substantial personal and social burden (2). However, the pathophysiology of migraine is complex and yet to be completely understood. There are many different pathophysiological hypotheses about the mechanism of migraine such as cortical spreading depression (CSD) (3), inflammation (4), subcortical dysfunction (5), and increase in oxidative stress (6). Consequently, a better understanding of migraine can play an important role in its treatment and prevention.

Proton magnetic resonance spectroscopy (^1^H-MRS) is a noninvasive method to detect brain metabolite concentrations and offers a better understanding of the underlying pathophysiologic mechanisms of migraine. Several studies have used ^1^H-MRS to investigate brain metabolite changes in migraineurs. However, their results were varied owing to the variable clinical inclusion criteria and methodology (7). The studies have reported increased (8–10) or decreased (11) glutamate, increased (12) or decreased (13, 14) gamma-aminobutyric acid, increased (12, 15) myo-inositol, decreased (11, 16) N-acetyl-aspartate, and decreased (17) choline. Furthermore, most of these studies have focused on migraine during the interictal phase; only three case reports have shown the results of brain metabolite changes in hemiplegic migraine during attacks, which is a rare variant of migraine with aura (18–20). Therefore, there is a lack of research on brain metabolites in patients with migraine during an attack, and filling this gap will enable us to better understand its mechanism.

The occipital lobe plays an important role in the pathophysiology of migraine, which is the original region of CSD (21). It is generally considered that CSD is the cause of the migraine visual aura because mapping the visual aura onto the occipital cortex is very similar to the temporal and spatial features of CSD (3). In addition, CSD is a putative migraine pain trigger, as it can activate the brainstem, which is involved in headache-inducing mechanisms (22, 23). Although CSD is closely related to aura, it is considered that CSD may also occur in migraine without aura (24), and both migraine with and without aura are linked to CSD (25). In addition, advanced neuroimaging studies have shown abnormal cortical activity (26, 27) and metabolite change (8, 9) in the occipital lobe of migraine without aura. Thus, in the present study, we chose occipital lobe as the region of interest to investigate the metabolite changes among patients with migraine without aura during attack (MWoA-DA), those with migraine without aura during the interictal period (MWoA-DI), and healthy controls (HC). We chose the occipital lobe, which is the key region of migraine, as the region of interest; moreover, it is the original region of CSD and is chosen by most studies (7). We also determined the relationship between metabolite levels and clinical scores to further explore the role of metabolite changes in migraine without aura.

## Materials and Methods

### Study Population

Patients with migraine without aura and HC were collected from the Department of Neurology at the First Affiliated Hospital of Zhejiang Chinese Medical University. All migraineurs fulfilled the diagnostic criteria of migraine without aura per the International Classification of Headache Disorders, third edition (ICHD-III) (1), and completed a headache questionnaire, which included medical history, disease duration, attack frequency (times/month), visual analog scale (VAS), Migraine Disability Assessment Scale (MIDAS), Headache Impact Test (HIT-6) scores, and Short-Form McGill Pain Questionnaire-2 (SF-MPQ-2) scores. Healthy controls were subjects who were free of other types of common headaches (e.g., tension type headache). None of the included subjects had contraindications to magnetic resonance imaging (MRI).

### Magnetic Resonance Image and Spectrum Acquisition

**Figure 1:**
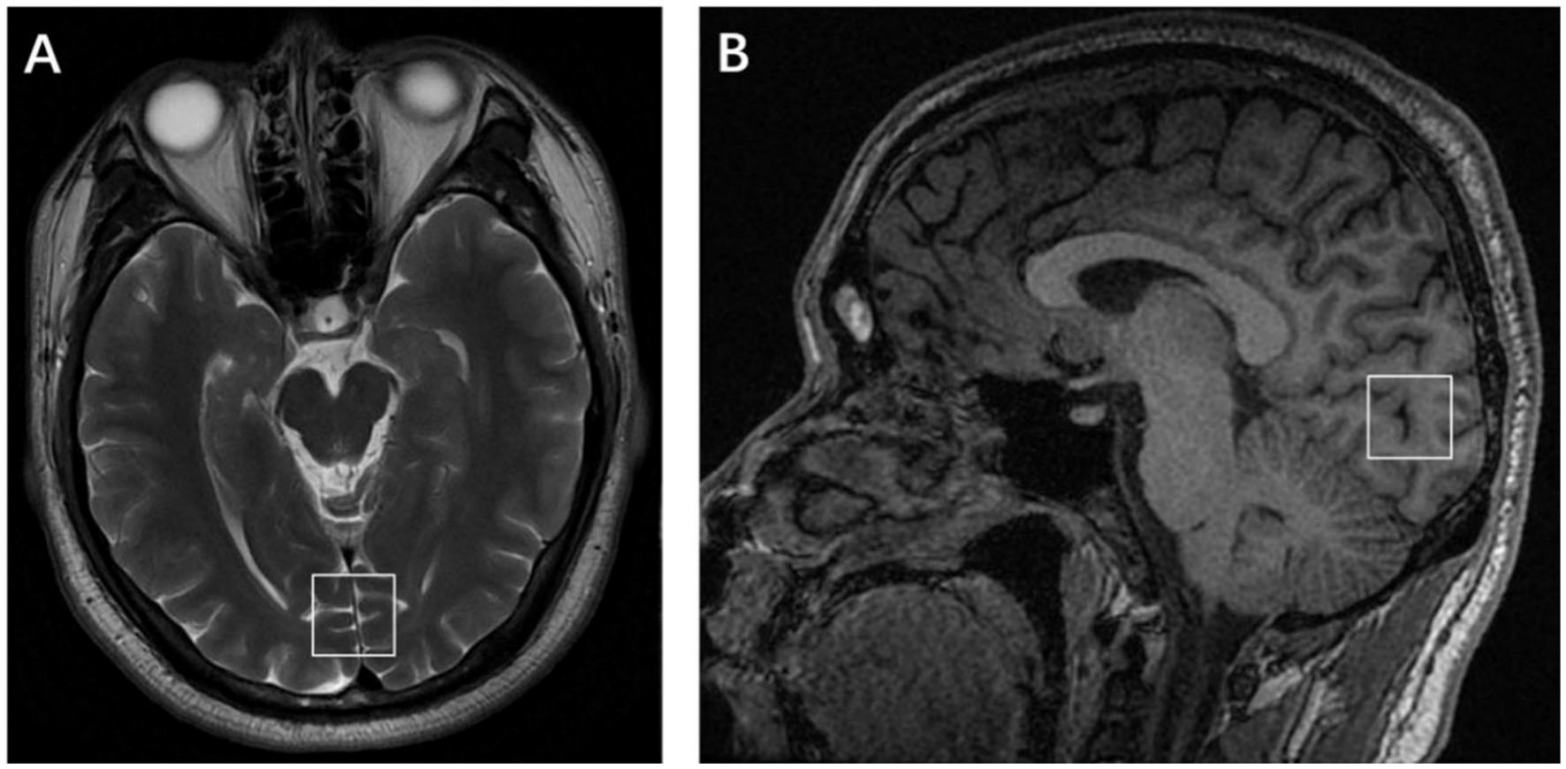
Placement of the single voxel in the axial **(A)** and sagittal **(B)** planes in the occipital lobe.

Subjects were scanned on a 3-Tesla GE Discovery MR750 scanner with an eight-channel head coil. The MRI session consisted of conventional T2-weighted image, T1-FLAIR, and DWI to make sure that participants had no intracranial lesions. A high-resolution T1-weighted scan (3D T1-BRAVO, TR = 8.2 ms, TE = 3.2 ms, flip angle = 12, matrix = 256 × 256, slice thickness = 1.0 mm with no gaps) was obtained and used for an accurate voxel placement. Single-voxel ^1^HMRS was performed with point-resolved spectroscopy (PRESS); volume of interest (VOI) was placed in the occipital cortex (Figure 1), TR = 2,000 ms, TE = 35 ms, voxel size = 20 × 20 × 20 mm^3^, total number of scans = 64, number of excitations (NEX) = 8, water suppressed, with automatic shimming. Pre-scan requirements were <8 Hz in automatic shimming at full width and at half maximum (FWHM) and >95% in water suppression.

### Magnetic Resonance Spectroscopy Data Processing

**Figure 2:**
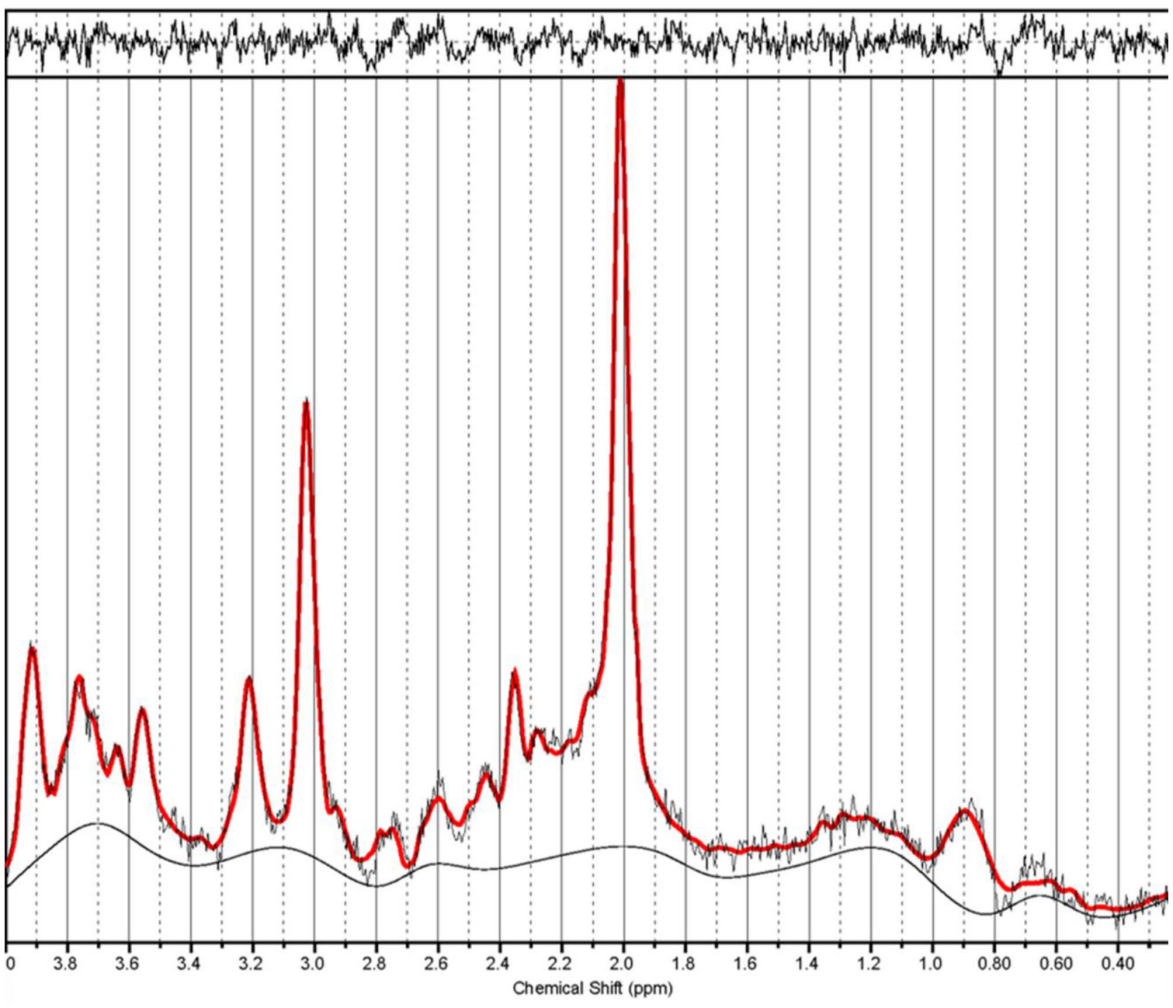
Example spectrum from a migraine without aura patient during the interictal period. The red line is the linear combination model (LCModel) fit to metabolite signals from this patient. The raw data are thin black lines that have the same shape as the red line. The thin black line at the bottom of the spectrum is the baseline.

The raw spectroscopy data was processed in linear combination model (LCModel) (Figure 2). N-acetyl-aspartate (NAA), glycerophosphocholine and phosphocholine (total choline, tCho), glutamate (Glu), creatine + phosphocreatine (total creatine, tCr), glutathione (GSH), and myo-inositol (MI) were measured in both patient and control groups. Because tCr was considered a reference metabolite and appeared stable in subjects, this study evaluated the relative concentration of metabolites as a ratio to tCr. Only metabolites with Cramér–Rao lower bounds <20% were considered reliable and included in further statistical analyses.

### Statistical Analysis

Statistical analyses were performed with the Statistical Package for Social Sciences software (SPSS 25.0). One-way analysis of variance (ANOVA) and Bonferroni-corrected *post hoc* analysis was performed to explore the difference between patients and controls. The relationship of clinical scores and metabolite ratios was assessed by Pearson's correlation coefficients. Statistical significance was set at *p* < 0.05.

## Results

### Demographic and Clinical Characteristics

Originally, we enrolled nine MWoA-DA, 14 MWoA-DI, and 13 HC. One patient with MWoA-DA and one with MWoA-DI were diagnosed with migraine with aura and, therefore, excluded. Two MWoA-DA patients were excluded for the following reasons: one patient used acute medication, and one had bad spectra quality CRLB higher than 20%. Finally, six MWoA-DA, 13 MWoA-DI, and 13 controls were included in this study. All migraineurs fit the criteria for episodic migraine, without medication overuse and had never taken migraine-preventive drugs during their life. The clinical characteristics for individual migraineurs are given in Table 1.

**Table 1:** Demographic and clinical data among three groups (mean ± SD).

	**MWoA-DA**	**MWoA-DI**	**HC**	***P*-value**
	**(n = 6)**	**(n = 13)**	**(n = 13)**	
Age (years)	39.17 ± 10.49	31.46 ± 9.03	31.85 ± 9.02	>0.05
Male/female	1/5	3/10	4/9	>0.05
VAS	7.67 ± 1.03	7.00 ± 1.52	–	
SF-MPQ-2	11.33 ± 1.75	8.77 ± 4.78	–	
MIDAS	43.83 ± 22.40	21.00 ± 14.36	–	
HIT-6	66.17 ± 4.26	60.46 ± 7.34	–	
Disease duration (years)	11.00 ± 2.82	6.71 ± 6.39	–	
Migraine frequency (days/month)	4.91 ± 3.58	3.23 ± 2.52	–	

*MWoA-DA, migraine without aura patients during attack; MWoA-DI, migraine without aura patients during interictal; HC, healthy controls; VAS, visual analog score; SF-MPQ, Short-Form McGill Pain Questionnaire-2; MIDAS, Migraine Disability Assessment; HIT-6, Headache Impact Test-6; –, not applicable*.

### Metabolite Changes

Overall, MWoA-DA patients showed a significantly lower GSH/tCr level than MWoA-DI and HC (*p* = 0.008 and *p* = 0.011, Bonferroni corrected, respectively) (Table 2 and Figure 3A). There were no statistical differences in GSH/tCr between the MWoA-DI and HC groups. The levels of tCho/tCr in MWoA-DA patients was lower than that in MWoA-DI and HC (*p* = 0.031 and *p* = 0.022, Bonferroni corrected, respectively) (Table 2 and Figure 3A). There were no statistical differences in tCho/tCr between the MWoA-DA and HC groups. There were no statistical differences in NAA/tCr, MI/tCr, and Glu/tCr among the three groups (all *p* > 0.05).

**Table 2:** Mean ratio of metabolite levels among three groups (mean ± SD).

	**MWoA-DA**	**MWoA-DI**	**HC**	***P*-value**	
	**(n = 6)**	**(n = 13)**	**(n = 13)**		
GSH	0.20 ± 0.02	0.27 ± 0.03	0.26 ± 0.04	0.008^*^	0.011^†^
tCho	0.16 ± 0.01	0.14 ± 0.01	0.16 ± 0.01	0.031^*^	0.022^#^
NAA	1.41 ± 0.11	1.37 ± 0.10	1.41 ± 0.12	>0.05	
Glu	1.38 ± 0.19	1.29 ± 0.09	1.26 ± 0.11	>0.05	
MI	0.71 ± 0.06	0.66 ± 0.10	0.66 ± 0.08	>0.05	

*MWoA-DA, migraine without aura patients during attack; MWoA-DI, migraine without aura patients during interictal; HC, healthy controls; GSH, glutathione; tCho, total choline; NAA, N-acetyl-aspartate; Glu, glutamate; MI, myo-inositol*.

* *MWoA-DA group compared with MWoA-DI group*.

† *MWoA-DI group compared with HC group*.

# *MWoA-DI group compared with HC group*.

**Figure 3:**
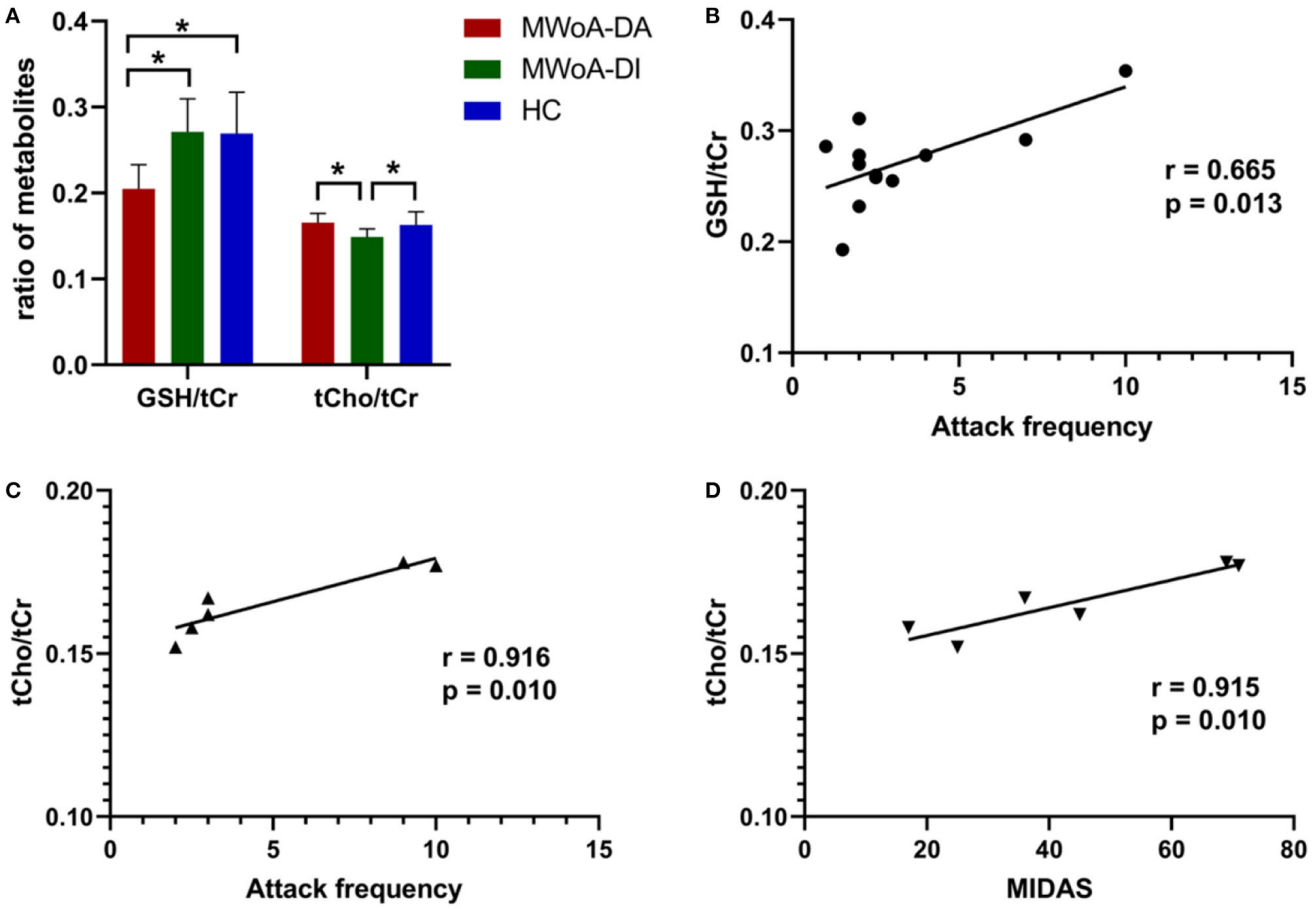
The mean ratio of brain metabolites of the three groups and correlation analysis. The GSH/tCr levels in the MWoA-DA group was lower than in the MWoA-DI and HC **(A)** groups. The tCho/tCr levels in the MWoA-DI group was lower than in the MWoA-DA and HC **(A)** groups. The levels of GSH/tCr of the MWoA-DI group correlated with the attack frequency **(B)**. The levels of tCho/tCr of the MWoA-DA group correlated with the attack frequency **(C)** and MIDAS **(D)**. GSH, glutathione; tCho, total choline; MWoA-DA, migraine without aura patients during attack; MWoA-DI, migraine without aura patients during interictal; HC, healthy controls; MIDAS, migraine disability assessment. *p < 0.05.

### Correlation Results

Within the MWoA-DI group, the GSH/tCr levels were correlated strongly with attack frequency (*p* = 0.013) (Figure 3B). The GSH/tCr levels were not associated with disease duration, VAS, MIDAS, HIT-6, and SF-MPQ-2 scores (all *p* > 0.05). Within the MWoA-DA group, tCho/tCr levels were correlated strongly with attack frequency and MIDAS scores (*p* = 0.010, 0.010; respectively) (Figures 3C,D). The tCho/tCr levels were not associated with disease duration, VAS, HIT-6, and SF-MPQ-2 scores (all *p* > 0.05).

## Discussion

In the present study, we used ^1^H-MRS to investigate the changes of brain metabolites in MWoA-DA and MWoA-DI subjects. The GSH/tCr levels were decreased only in MWoA-DA patients, while the tCho/tCr levels were only decreased in MWoA-DI patients.

Several studies have used phantom data to provide evidence that it is possible to measure changes in GSH concentration using ^1^H-MRS, and GSH can be quantified reproducibly *in vivo* (28, 29). GSH has been measured by ^1^H-MRS in humans in a number of neurological diseases such as Alzheimer's disease (30), schizophrenia (31), and epilepsy (32). To our knowledge, this study was the first to investigate the changes in brain GSH in migraineurs using ^1^H-MRS. GSH is the major cellular antioxidant that is essential for cellular function, plays roles in oxidation–reduction reactions, and protects against reactive oxygen species (33, 34). Our study found decreased GSH/tCr levels in the occipital lobe of MWoA-DA patients suggesting enhanced oxidative stress and a depletion of antioxidant defenses during the migraine attack; thus, the decreased GSH/tCr levels is the result, rather than the cause, of migraine attacks, as there was no difference between MWoA-DI and HC. Our results were consistent with those of a study that found that oxidative stress was elevated during migraine attacks, but not interictally (35). Oxidative stress plays a major role in migraine pathophysiology (36). It is generally believed that most migraine triggers or aggravating factors have a link to energy metabolism and oxidative stress (6, 37). Reasonable research has shown increased oxidative stress parameters and decreased anti-oxidative parameters in migraine patients (38–40). In addition, the prophylactic use of antioxidants can significantly reduce migraine frequency and pain intensity (36). Our study provides ^1^H-MRS-based evidence of oxidative stress playing a role in the pathophysiology of migraine during attack.

Correlation results have shown a significant positive relationship between GSH/tCr and attack frequency in the MWoA-DI group. Unlike depletion in GSH/tCr levels due to oxidative stress in the MWoA-DA group, GSH/tCr in the MWoA-DI group showed no difference with respect to HC. Furthermore, MWoA patients with higher attack frequency tended to have higher GSH levels in the interictal phase. Our result is similar to one study that found that glutathione peroxidase-1 levels were positively correlated between attack frequency (41). GSH is the major cellular antioxidant, and plays a critical role in maintaining neuronal health (33, 34). Therefore, we speculate that GSH positively correct with attack frequency in the MWoA-DI group may be the result of self-protective mechanism of the body. Moreover, GSH is compensatorily increased to prevent migraine attacks in the future, and the more serious the disease, the higher is the GSH concentration.

In the present study, reduced tCho/tCr levels were found in the occipital lobe of MWoA-DI. However, the tCho/tCr levels were returned to normal during the attack. This result likely shows the existence of different pathophysiological states between MWoA-DA and MWoA-DI patients. In the brain, Cho resonance detected by MRS is mainly from phosphocholine and glycerophosphocholine. Therefore, Cho represents the metabolism of the cell membrane and membrane turnover (phospholipid synthesis and degradation). A previous study also reported a decreased Cho/Cr level in the cerebellum of migraineurs, suggesting alterations in membrane composition (17). Altered Cho signal may imply the changes in membrane turnover and thus suggest altered neuronal integrity and functional impairment (42). Therefore, the decreased tCho/tCr levels found in our study may suggests neuronal dysfunction in MWoA-DI patients.

Nowadays, neuronal dysfunction is thought to be a predisposing factor to migraine attack onset (43). It is generally believed that migraineurs have mitochondrial dysfunction and impairment of the brain energy metabolism, which has a lower energy reserve and velocity of oxidative metabolism (7, 44). Several resting-state functional magnetic resonance imaging studies have also found that the indicator of cortical activity and neural synchronous were lower in the occipital cortex of the MWoA-DI group (26, 27). Therefore, our result may provide useful evidence for the hypothesis that neuronal dysfunction serves as the predisposing factor for migraine attack onset.

Interestingly, tCho/tCr levels returned to normal during the attack; in other words, they were relatively increased compared with that found in MWoA-DI patients in the present study. Increased tCho/tCr is related to the activation or proliferation of glial cells, which will be leading to abnormal phospholipid metabolism and accelerated cell membrane turnover (45–47). In addition, activation of glial cells is a well-known sign of inflammation in the brain (48). Thus, we think this result is probably linked to a compensation mechanism induced by ictal neuroinflammation. We suspected that the inflammation suggested in our experiment might be due to CSD or oxidative stress. CSD is often accompanied with inflammation, and it can increase the number and volume of astrocytes as well as the expression of marker proteins of inflammation (49). Though the patients in this study were migraineurs without aura, it is considered that CSD may also occur in migraine without aura (24) and likely play a role in the pathophysiology of migraine without aura (25, 50). However, more research is needed to further explore this topic. Another factor that causes inflammation may be oxidative stress. It is considered that inflammation can be triggered and amplified by oxidative stress and play an important role in the pathogenesis of acute pain arising from migraine (38, 39). Furthermore, there were significant positive correlations between tCho/tCr and attack frequency and MIDAS scores in MWoA-DA patients. This suggests that the more severe the migraine, the more intense the inflammation during a migraine attack. These observations suggest that neuroinflammation may be involved in the pathophysiology of migraines.

The current study has some limitations. First, the sample size of this study was rather small. Therefore, these results need further replication with a large sample size. Second, this study was a cross-sectional study, and although we confirmed that patients had no migraine attacks within 48 h before and after the MRI scanning, we do not know what day of the migraine cycle the patient was in on the day of the scan. Further studies are needed to carry out longitudinal analysis to dynamically observe migraine brain metabolite changes. Third, in this study, GSH/tCr was quantified without the use of spectral editing. Although studies suggest that non-editing techniques are comparable or better than a spectral editing technique (28), further studies are needed to quantify GSH/tCr use-specific editing spectral.

## Conclusion

To our knowledge, this study was the first to investigate the changes in brain GSH/tCr levels in migraineurs using ^1^H-MRS and the first to investigate the changes in brain metabolites in MWoA-DA patients. We found decreased tCho/tCr levels in MWoA-DI patients, decreased GSH/tCr levels, and relatively increased tCho/tCr levels in migraine during attack. These findings may indicate that neuronal dysfunction may serve as the predisposing factor to migraine attack onset, and oxidative stress and inflammation may take part in the migraine attack. These findings contribute to the understanding of migraine.

## Data Availability Statement

The raw data supporting the conclusions of this article will be made available by the authors, without undue reservation.

## Ethics Statement

The studies involving human participants were reviewed and approved by Ethics Committee of the first Affiliated Hospital of Zhejiang Chinese Medical University (2018-KL-081-02). Due to the retrospective study, informed consent was waived.

## Author Contributions

ZC and ZZ contributed to the study design and clinical data collection. LZ and JH performed the experiments and analyzed the data. LZ prepared the manuscript. All authors read and approved the final manuscript.

## Conflict of Interest

The authors declare that the research was conducted in the absence of any commercial or financial relationships that could be construed as a potential conflict of interest.
